# Linking ADHD to Depression in Adolescents: the Mediating Role of Social Skills

**DOI:** 10.1007/s10802-026-01430-5

**Published:** 2026-03-02

**Authors:** Allison M. Loomis, Sierra R. Hightower-Henson, Steven W. Evans, George J. DuPaul

**Affiliations:** 1 Department of Psychology, The Ohio State University, Columbus OH, USA; 2 Department of Psychology, Ohio University, Athens OH, USA; 3 Institute for Mental Behavioral Health Research, Nationwide Childrens Hospital, Columbus OH, USA; 4 Department of Psychiatry Behavioral Health, The Ohio State University, Columbus OH, USA; 5 Department of Education and Human Services, Lehigh University, Bethlehem PA, USA

**Keywords:** ADHD, Depression, Social skills, Mediation, Adolescence

## Abstract

Adolescents with attention-deficit/hyperactivity disorder (ADHD) are at a higher risk for developing depression than their peers. One potential explanation for this increased risk is that adolescents with ADHD often have impaired social skills, which may interfere with successful social interactions and connections, and subsequently lead to low self-worth and depression. We hypothesized that global social skills and social skill factors most related to friendships (communication, cooperation, engagement, and empathy) would mediate the positive relation between ADHD symptom severity and depression. Additionally, we investigated whether global social skills would mediate the relations between ADHD symptom severity and four facets of depression (negative self-evaluation, anhedonia, dysphoric mood, and somatic complaints). Our sample included 335 adolescents with ADHD in grades 6–11 who participated in an intervention study. Parent-reported ADHD symptoms were measured at or before baseline, parent-reported social skills (global and several subscales) were measured one year later, and self-reported depression was measured 1.5 years after baseline. Global social skills mediated (1) the relation between ADHD and depression and (2) the relation between ADHD and negative self-evaluation. Cooperation, communication, empathy, and responsibility individually mediated the relation between ADHD and depression, and assertion, self-control, and engagement did not.

**Supplementary Information:**

The online version contains supplementary material available at 10.1007/s10802-026-01430-5.

## Introduction

Adolescents with attention-deficit/hyperactivity disorder (ADHD) have higher rates of depression than their peers without ADHD, with approximately 14% of adolescents with ADHD, as opposed to 8% of all adolescents, meeting the criteria for major depressive disorder (MDD; Daviss, 2008; Shorey et al., 2022; Turgay et al., 2005). Depression during adolescence is positively associated with a variety of negative outcomes, including suicide attempts, aggression, and poor adjustment in academic, behavioral, and social domains (Slomkowski et al., 1995). Because both ADHD and depression are independently associated with a variety of adverse outcomes (Daviss, 2008; Treuting & Hinshaw, 2001), youth experiencing both disorders are at serious risk for multiple poor long-term outcomes. Explanations for the increased risk of depression among youth with ADHD include genetics (Riglin et al., 2021), poor long-term academic struggles (Arnold et al., 2020), and social impairment (Schoeler et al., 2018). Of these, researchers have given considerable attention to exploring types of social functioning as potential mediators of the relation between ADHD and depression (e.g., parent-adolescent conflict and peer relationships; Eadeh et al., 2017; Powell et al., 2020).

Social impairment is common among adolescents with ADHD, as they often have fewer friendships and are less involved in social activities than their non-ADHD peers (Bagwell et al., 2001). Social impairment can lead to feelings of failure and isolation and, consequently, may lead to low self-worth and depression (Capaldi, 1992). Although a few studies examined the role of social functioning in the relation between ADHD and depression (Humphreys et al., 2013; Powell et al., 2020; Roy et al., 2015), these studies did not focus on the aspects of depression (e.g., negative self-evaluation, anhedonia) that may be most impacted by ADHD. Identifying such aspects may help us improve our understanding of the comorbidity between ADHD and depression and recognize the aspects of depression that should be prioritized during intervention for adolescents with ADHD. Also, these studies did not consider the potential role of various aspects of social skills (e.g., communication and empathy) to explain the relation between ADHD and components of depression. Understanding the relations between types of social skills, ADHD, and characteristics of depression can help us understand the relative risk of pre-pubertal youth for developing depression and include implications for treatment. Therefore, the purpose of the present study was to examine the role of social functioning, including various social skill factors, in the development of depression and its facets among adolescents with ADHD.

### ADHD and Depression

Multiple studies have suggested that adolescents with ADHD are at risk for developing depression during adolescence and early adulthood (Meinzer et al., 2013, 2016). Thus, researchers have examined many characteristics to see if they mediate, or explain, the relation between ADHD and depression. However, evidence suggests that other internalizing and externalizing disorders, such as anxiety, conduct problems, and aggression, do not fully account for this relation (Blackman et al., 2005; Biederman et al., 2008; Herman et al., 2007). Additionally, recent work highlights that ADHD has unique effects on affective symptoms independent of other disruptive behavior diagnoses, such as oppositional defiant disorder (ODD; Ágrez et al., 2025). Academic difficulties have also been implicated, with some support for academic performance mediating the link between ADHD and later depressive symptoms (Herman et al., 2007). Yet other studies indicate that this pathway does not hold once accounting for poor social functioning (Eadeh et al., 2017; Humphreys et al., 2013) and parent-adolescent conflict (Humphreys et al., 2013). Thus, these findings suggest that social impairments may be more central to the development of depression in adolescents with ADHD than academic functioning.

Social impairment has also been consistently highlighted as a contributor to the development of depression in adolescents with ADHD because experiences of failure in a social context (e.g., having fewer relationships, being less involved in social activities, experiencing peer rejection) may influence self-evaluation (Becker et al., 2017; Dvorsky et al., 2019). These difficulties predict both depression and low self-worth in adolescents (Becker et al., 2017; Dvorsky et al., 2019; Eberhart & Hammen,  2006; Platt et al., 2013), and longitudinal research shows that peer problems, friendship quality, and social competence partially mediate the relation between ADHD and depression (Humphreys et al., 2013; Powell et al., 2020; Roy et al., 2015). Nonetheless, these studies (1) did not consider the aspects of depression that may be most impacted by social impairment, such as negative self-evaluation, and (2) relied on parent, teacher, and peer ratings of broad peer relationship quality and social functioning as measures of adolescents’ social impairment, without the consideration of specific social skills, such as communication or empathy.

In addition to examining components of social functioning and depression, it is important to consider the role of the two symptom factors of ADHD when focusing on adolescents. Adolescents typically exhibit fewer hyperactivity/impulsivity symptoms than they did in childhood (Sibley et al., 2012), and inattention has been found to be a stronger predictor of social impairment, social withdrawal, and internalizing symptoms than hyperactive/impulsive symptoms (Willcutt et al., 2012; Zoromski et al., 2015). In line with this finding, Humphreys and colleagues (2013) noted that inattention, but not hyperactivity, predicted peer and parent-child problems, and these problems mediated the relation between inattention and depression. Taken together, these findings suggest that researchers need to prioritize the role inattentive symptoms have with social impairment, as evidence suggests that inattention may be the primary aspect of ADHD that is associated with social functioning. Therefore, we evaluated both inattentive and hyperactive/impulsive symptom severity separately in our models to determine whether it is primarily inattention that predicts social skills functioning and depression development, or whether both symptom factors (i.e., ADHD symptoms overall) are predictive of these outcomes.

### Facets of Depression

Although ADHD and depression can be comorbid, it remains unclear which aspects of depression are most impacted by ADHD and social skills functioning. The Reynolds Adolescent Depression Scale**-**Second Edition (RADS-2; Reynolds, 2002) is a common measure of depression experienced by adolescents, and it consists of four subscales: Anhedonia/Negative Affect (e.g., lack of the ability to experience pleasure), Negative Self-Evaluation (e.g., feeling worthless), Somatic Complaints (e.g., troubles sleeping and feeling sick), and Dysphoric Mood (e.g., feeling sad). Of these, negative self-evaluation may be especially relevant for adolescents with ADHD. Unlike global self-worth, which tends to be more stable, feelings of self-worth (i.e., self-evaluation) may rise and fall in response to certain events (Brown et al., 2001; Harter, 2012). Competency-based theories suggest that repeated academic and social difficulties undermine self-worth (Mrug et al., 2012), and ADHD symptoms may manifest or be interpreted as avoidance and procrastination, which may also contribute to these negative appraisals (Knouse et al., 2023). The vulnerability model of depression proposes that low self-worth is not only a characteristic of depression but also that low self-worth is a personality characteristic that predisposes people to develop depression (Klein et al., 2011), which is consistent with evidence that self-worth is concurrently and longitudinally related to depression (King et al., 1993; McQuade et al., 2011). Given that low self-worth may be a precursor to depression among adolescents and that negative self-evaluation involves feelings of self-worth that are particularly reactive to negative social experiences, research on the emergence of depression in youth with ADHD should be expanded to include negative self-evaluation, especially in young adolescents.

One previous study utilizing some of the same data used in this study by Becker and colleagues (2015) investigated which facets of depression were significantly associated with self-reported and parent-reported social skills. They found that while Anhedonia was the only depression facet significantly associated with self-reported social skills, Negative Self-Evaluation was the only depression facet significantly associated with parent-reported social skills. Explanations for these findings could be that parents are especially attuned to signs of worthlessness and helplessness, and peer rejection and fewer close friendships may directly erode adolescents’ feelings of self-worth (Becker et al., 2017; Dvorsky et al., 2019). However, this work did not test whether social skills impairment leads to negative self-evaluation. Building upon these findings, it is important to examine the extent to which social functioning explains the development of negative self-evaluation, as well as other facets of depression, in adolescents with ADHD.

### Types of Social Skills

A few studies established social functioning as a mediator between ADHD and depression (Humphreys et al., 2013; Powell et al., 2020; Roy et al., 2015). Adolescents with ADHD often have fewer reciprocal friendships and experience higher levels of peer rejection than their peers (Bagwell et al., 2001), and such impairments are robust predictors of depression (Eberhart & Hammen, 2006; Platt et al., 2013). These social impairments are a function of many aspects of the context as well as the social skills of the teen. By identifying which skills are associated with the impairment and depression, it will be possible to hone the precision of interventions targeting social impairment by clarifying the skill deficits that are important to target. Currently, it remains unclear which specific social skills (e.g., communication, cooperation, responsibility, engagement, assertion, empathy, and self-control) most significantly contribute to the relation between ADHD and depression. Social skill deficits, including disruptive and inappropriate social behaviors, are a prominent outcome of ADHD and are related to internalizing psychopathology (Gardner & Gerdes, 2015; Morgan et al., 2022). Therefore, social skills are likely a promising area of social impairment to target with adolescents with ADHD. Identifying the social skills that contribute most to self-worth and depression can add precision to our understanding of these relationships and inform the choice of targets for intervention development.

Studies suggest that crucial characteristics of friendships during adolescence include self-disclosure, intimacy, communication, and engaging in activities together (Buhrmester, 1990; Mannarino, 1976), and empathy may play a key role in the development of prosocial behavior (Marshall et al., 2019). In contrast, there is less support in the developmental literature that the other social skills factors (Self-Control, Responsibility, and Assertion) play a central role. These findings, in combination with the social skills that most closely map onto the Diagnostic and Statistical Manual of Mental Disorders (5th ed., text rev.; DSM-5; American Psychiatric Association, 2022) criteria for depression, were used to generate hypotheses of which social skills would significantly mediate the link between ADHD and depression. Specifically, the DSM-5 depression criteria of “diminished ability to think or concentrate,” “fatigue or loss of energy,” “diminished interest or pleasure in most activities,” and emotional blunting (an aspect of psychomotor slowing) were used to hypothesize that engagement, communication, cooperation, and empathy would significantly correlate with depression and mediate the link between ADHD and depression, and the other social skills (i.e., responsibility, assertion, and self-control) would not.

### Present Study

Adolescents with ADHD frequently experience social impairment, including having fewer friendships and receiving more peer rejection than their non-ADHD peers (Bagwell et al., 2001). Research suggests that these social impairments are associated with depression and low self-worth (Dvorsky et al., 2019; Platt et al., 2013) and that social skills impairment may mediate the relation between ADHD and depression (Humphreys et al., 2013; Powell et al., 2020; Roy et al., 2015). However, questions persist about the facets of depression that are most impacted by ADHD, such as negative self-evaluation and anhedonia. Previous research suggested that fewer prosocial skills and negative peer relationships are associated with low self-worth and that negative self-evaluation and anhedonia may be related to social skills in adolescents with ADHD (Becker et al., 2015, 2017; Scharf & Mayseless, 2009). Therefore, our first research question is focused on identifying the extent to which social skills mediate the positive relations between (1) ADHD symptom severity and depression and (2) ADHD symptom severity and the facets of depression (e.g., negative self-evaluation, anhedonia).

It also remains unclear what types of social skills significantly explain the relation between ADHD and depression, as previous studies focused on the mediating roles of global social impairment, peer rejection, or peer and family relationships. To better inform future prevention efforts and understand the progression of depression, it is important to identify the individual social skills that mediate the relation between ADHD and depression. Developmental literature suggests that the social skills crucial for forming and sustaining friendships, including communication, cooperation, empathy, and engagement, may be most relevant to depression and self-worth. Therefore, as a second research question, we sought to determine which types of social skills most significantly contribute to the relation between ADHD symptom severity and depression.

## Methods

### Participants

The sample consisted of 335 middle and high school students (23.9% sixth, 21.8% seventh, 18.8% eighth, 15.8% ninth, 13.4% tenth, and 6.3% eleventh grade) diagnosed with ADHD. The sample included those who participated in a school-based randomized control trial for an intervention targeting adolescents with ADHD and completed all measures in our hypothesized mediation models (e.g., ADHD symptoms at T1, social skills at T2, depression at T3). Participants ranged from age 10 to 16 (*M* = 12.75, *SD* = 1.65) and included 247 (73.7%) boys and 88 (26.3%) girls. From an initial sample size of 801, 264 (32.96%) had missing data at T1, 386 (48.19%) had missing data at T2, and 433 (54.06%) had missing data at T3, resulting in a final sample size of 335 (41.82% of the original sample). We conducted *t*-tests for the main variables in our model for participants missing data vs. those without any missing data. Participants without missing data had statistically significantly higher average levels of ADHD symptom severity (*M* = 19.24, *SD* = 5.47) than participants with missing data (*M* = 17.91, *SD* = 5.70), but this difference was practically small (*d* = − 0.24). Social skills at T2 and depression at T3 did not statistically significantly differ for participants with and without missing data.

Out of the 329 students with race data, 79.0% were White, followed by 11.3% Black, 0.6% Asian, 0.3% Native Hawaiian or Pacific Islander, 0.3% Native American or Alaskan Native, and 8.5% multi-racial or other. Out of the 284 with ethnicity data, 95.1% of the students were not Hispanic or Latinx, and 4.9% were Hispanic or Latinx.

### Procedure

Research procedures for the randomized trials conducted to collect these data were approved by Ohio University’s institutional review board (IRB), IRBs at collaborating institutions, and school administrators. IRB approval was not obtained for this specific study as only de-identified data were used in these analyses. Participants were recruited from middle schools at a separate time than those recruited from high schools although recruitment and evaluation procedures were very similar to each other. Youth were recruited from rural, suburban, and urban middle and high schools in Ohio and Pennsylvania. Consent from parents and assent from youth were obtained from all participants at the initial eligibility evaluations. All students were screened and diagnosed with ADHD and ODD using the Parent Children’s Interview for Psychiatric Syndromes (P-ChIPS; Weller et al., 2000) or through parent and teacher reports using the Disruptive Behavior Disorders Rating Scale (Pelham et al., 1992) or the ADHD Rating Scale 5 (DuPaul et al., 2016). Additionally, no participants met the criteria for bipolar disorder, psychosis, or obsessive-compulsive disorder based on the administration of P-ChIPS. Middle school students additionally did not meet the criteria for pervasive developmental disorder and substance dependence (except for tobacco), and high school students did not meet the criteria for pervasive development disorder or exhibit problematic substance use (Subtle Screening Inventory, Adolescent, Second Edition; Miller & Lazowski, 2001).

Students completed assessments at multiple time points throughout the academic year. Eligibility assessments (T1) were completed from March to August prior to the start of the school year. Mid-year assessments (T2) were completed from January to March, and assessments were also administered at the end of the year in May (T3).

Despite many similarities, the middle and high school samples were collected from studies done at two different times with different priorities. The primary differences between the two samples involve IQ, the RADS-2, and treatment groups.

#### IQ

Participants had an IQ of at least 80 (middle school) or 75 (high school) as determined by the Wechsler Intelligence Scale for Children – Fourth Edition (Wechsler, 2003; middle school) or the Wechsler Abbreviated Scale of Intelligence – Second Edition (Wechsler, 2011; high school). The IQ cutoff was lowered in the high school study to increase recruitment and generalizability. Although there was a statistically significant difference in IQ scores between middle (*M* = 95.09, *SD* = 12.73) and high (*M* = 98.38, *SD* = 14.24) school students (*t* = −2.17, *p* =.03), this difference is equivalent to the standard error of the test and not meaningful.

#### RADS-2

To reduce participant burden, the short form of the RADS-2 was used for the high school sample. Previous studies found very high correlations between the short and full-length forms of the RADS-2 in adolescents (Milfont et al., 2008, *r* =.95; Ortuño-Sierra et al., 2017, *r* =.91). Therefore, there are minimal differences between scores in the full-length and short forms.

#### Treatment Groups

Students were recruited during the spring using flyers distributed in schools, letters sent to families, and referrals from school staff. Middle school students were randomly assigned to one of three treatment conditions, including an after-school treatment program, mentoring intervention, or a community care condition (Evans et al., 2016). High school students were assigned to either a treatment or a community care condition (Evans et al., 2024). All conditions were included in the present study, and condition was added as a covariate in all models.

### Measures

#### ADHD Symptom Severity

We chose to rely on parent ratings of ADHD symptoms given the limitations of secondary school teacher ratings of this construct (Evans et al., 2005; Molina et al., 1998).

##### Disruptive Behavior Disorders Rating Scale (DBD; Pelham et al., 1992)

Parents completed the DBD at baseline (T1) to measure middle school students’ ADHD symptoms. The inattentive (α = 0.88) and hyperactive/impulsive (α = 0.89) subscales were used, which include nine items each reflecting DSM-III ADHD symptoms that use a four-point Likert scale ranging from *0 (not at all)* to *3 (very much).* Total inattention and hyperactivity/impulsivity symptom severity scores each range from 0 to 27, with a higher score indicating higher symptom severity. The DBD has demonstrated strong internal consistency in samples of children and adolescents (α = 0.95) and is strongly associated with behavioral measures and clinical diagnoses of ADHD, providing support for its validity (Pelham et al., 1992; Wright et al., 2007).

##### ADHD Rating Scale-5 (ARS-5; DuPaul et al., 2016)

Parents completed the ARS at baseline (T1) to measure high schoolers’ ADHD symptoms. The inattentive (α = 0.86) and hyperactive/impulsive (α = 0.89) subscales were used, which include nine items each reflecting DSM-5 ADHD criteria (the same symptoms included in the DBD for middle school students) that use a four-point Likert scale ranging from *0 (not at all)* to *3 (very much).* Total inattention and hyperactivity/impulsivity symptom severity scores each range from 0 to 27, with a higher score indicating higher symptom severity. The ARS-5 has demonstrated internal consistency and construct validity in child and adolescent samples with a hierarchal omega of 0.85 using the DSM-5’s two-factor structure (DuPaul et al., 2016).

#### Social Skills

##### Social Skills Improvement System – Rating Scale (SSIS-RS; Gresham & Elliott, 2008) 

Parents completed the SSIS-RS at mid-year (T2) for both middle and high school students to measure how likely their child is to participate in various social behaviors. The SSIS-RS contains 46 items with seven social skills subdomains. These subdomains include Communication, Cooperation, Assertion, Responsibility, Empathy, Engagement, and Self-Control. Each subscale contains six to seven items that use a four-point Likert scale ranging from *0 (never)* to *3 (almost always).* Total social skills scores range from 0 to 138, with a higher score indicating more use of positive social skills and a lower score indicating higher social skills impairment. The SSIS-RS social skills scores demonstrated strong internal consistency in samples of elementary (α = 0.95) and secondary (α = 0.96) aged students and the scores are strongly associated with the Social Skills Rating System (SSRS; Gresham & Elliott, 1990; Gresham et al., 2011). We used total socials skills raw scores (middle school α = 0.94; high school α = 0.94) and each subdomain (middle school: Communication α = 0.72, Cooperation α = 0.81, Assertion α = 0.68, Responsibility α = 0.84, Empathy α = 0.90, Engagement α = 0.79, Self-Control α = 0.83; high school: Communication α = 0.75, Cooperation α = 0.84, Assertion α = 0.57, Responsibility α = 0.86, Empathy α = 0.86, Engagement α = 0.79, Self-Control α = 0.82) in our analyses. Note that the SSIS-RS reliabilities were calculated for the entire initial sample (rather than only the 335 participants with complete data for our model) since participant ID numbers were removed for item-level data.

#### Depression

##### Reynolds Adolescent Depression Scale, Second Edition (RADS-2; Reynolds, 2002)

Middle school students self-reported depression symptoms using a Likert scale ranging from *1 (almost never)* to *4 (most of the time).* The RADS-2 consists of 30 items that assess four factors of depression, including Dysphoric Mood, Negative Affect, Negative Self-Evaluation, and Somatic Complaints. Total depression symptom severity scores range from 30 to 120, with higher scores indicating more severe depression symptoms. However, T-scores were used to interpret mean scores and combine the data with the high school sample. Data from time point T3 were used in the model and T1 scores were used as covariates. The RADS-2 has demonstrated strong internal consistency and test-retest reliability in samples of children and adolescents (Reynolds, 2002).

##### Reynolds Adolescents Depression Scale- Short Form (RADS-2-SF; Reynolds, 2008)

High school students self-reported depression symptoms using a Likert scale ranging from *1 (almost never)* to *4 (most of the time).* The RADS-2-SF consists of 10 items that assess one global depression factor. Total scores range from 10 to 40, with higher scores indicating more severe depression symptoms. However, T-scores were used to interpret means and combine the data with the middle school sample. Data from time point T3 were used in the model (α = 0.91), and T1 scores were used as covariates (α = 0.86). The RADS-2-SF has been shown to have convergent validity with other measures of depression and to be highly correlated with the full-length RADS in an adolescent sample (Ortuño-Sierra et al., 2017).

### Data Analytic Plan

Descriptive statistics and bivariate correlations were calculated for all measures, including each type of social skill. A Bonferroni correction was used since we evaluated multiple comparisons. To evaluate the extent to which social skills mediated the relation between ADHD and depression, we first tested two mediation models using the PROCESS macro Model 4 (Hayes, 2022) on SPSS v. 29.0.2, with depression symptom severity at T1, treatment condition, ODD diagnosis, and age at T1 entered as covariates. The models included ADHD symptom severity (Model 1: inattentive; Model 2: hyperactive/impulsive) at T1 as an explanatory variable, total social skills at T2 as the mediating variable, and depression symptom severity at T3 as the outcome variable. Ten thousand bootstrap samples were used to estimate indirect effects with 95% bootstrap confidence intervals. Since both models indicated a similar significant indirect effect and the two symptom factors were highly correlated (*r* =.48), we reported this model and all further analyses using combined ADHD symptom severity.

To explore which facets of depression were significant outcomes of ADHD, with social skills mediating their relation, the same model was tested in middle school students (with whom the full RADS-2 was used) with Anhedonia, Negative Self-Evaluation, Dysphoric Mood, and Somatic Complaints as outcomes. As before, these models included the depression facet at T1, treatment condition, ODD diagnosis, and age at T1 as covariates. Given the exploratory approach of this second research question, a correction for multiple comparisons was not included.

Lastly, to evaluate which types of social skills significantly explain the relation between ADHD and depression, individual social skills factors were entered as mediators between ADHD symptom severity at T1 and depression at T3 with the full sample of middle and high school students, including the same covariates as the total social skills model.

## Results

Descriptive statistics of demographics and all variables included in the models are reported in Table 1. On average, adolescents exhibited high levels of ADHD symptom severity at TI, as reported by parents (*M* = 30.43, *SD* = 10.49). In addition, adolescents had non-clinical (T-score < 60) levels of depression symptom severity on average at T1 (*M* = 45.44, *SD* = 9.43) and T3 (*M* = 45.51, *SD* = 10.72). The similarity between depression scores at T1 and T3 is likely due to the short time frame of the study, as these time points were an average of only 1.5 years apart. It is common for depression symptoms to be stable during this time frame (Holsen et al., 2000). Within our sample, 19.7% met criteria for ODD. Youth who were not diagnosed with ODD had significantly lower levels of depression at T1 (*M* = 44.88, *SD* = 9.16) than those with ODD (*M* = 47.70, *SD* = 10.25; *t*(333) = −2.19, *p* =.03), although the difference was small (*d* = − 0.30). At T3, youth without (*M* = 45.57, *SD* = 10.60) and with (*M* = 45.27, *SD* = 11.26) ODD did not significantly differ in depression symptom severity (*t*(333) = 0.20, *p* =.84).

**Table 1:** Descriptive statistics of demographics and variables in models

	*n*	Freq (%)	*Min*	*Max*	*M*	*SD*
Sex (*N* = 335)						
Male	247	73.73				
Female	88	26.27				
Grade (*N* = 335)						
6th	80	23.88				
7th	73	21.79				
8th	63	18.81				
9th	53	15.82				
10th	45	13.43				
11th	21	6.27				
Race (*N* = 329)						
White	260	79.03				
Black or African American	37	11.25				
Asian	2	0.61				
Native Hawaiian/Pacific Islander	1	0.30				
Native American/Alaskan Native	1	0.30				
More than one race/other	28	8.51				
Primary Parent Income (*N* = 328)			1	9	4.72	1.91
Parent 1 Education (*N* = 317)			1	6	4.10	1.09
Parent 2 Education (*N* = 263)			1	6	3.87	1.13
Age (*N* = 335)			10	16	12.75	1.65
Condition (*N* = 335)						
Treatment	141	42.1				
Mentoring Intervention (MS only)	72	21.5				
Control (Community Care)	122	36.4				
ADHD (T1; DBD/ARS-5; *N* = 335)			4	54	30.43	10.49
Social Skills (T2; SSIS-RS; *N* = 335)			43	127	85.84	16.43
Communication			3	21	14.04	3.38
Cooperation			2	18	10.65	3.21
Assertion			3	20	12.93	3.03
Responsibility			1	18	10.94	3.49
Empathy			1	18	11.27	3.67
Engagement			3	21	12.56	3.59
Self-Control			1	21	10.49	3.80
Depression (T1; RADS-2; *N* = 335)			30	77	45.44	9.43
Depression (T3; RADS-2; *N* = 335)			29	83	45.51	10.72
Negative SE (T3; RADS-2, *N* = 215)			39	81	47.41	9.24
Anhedonia (T3; RADS-2; *N* = 216)			38	91	50.00	9.30
Dysphoric Mood (T3; RADS-2; *N* = 216)			29	78	44.58	9.57
SC (T3; RADS-2; *N* = 216)			28	75	44.19	10.82

Primary Parent Income = 1, up to 10,001–14,999; 3, 25,000–49,999; 5, 75,000–99,999; 7, 150,000–199,999; 9, $200,000 or more. Parent Education = 1, less than 9th; 2, partial high school; 3, high school diploma or equivalent; 4, some college/associate degree; 5, bachelor’s degree; 6, graduate degree. MS = middle school. DBD = Disruptive Behavior Disorders Rating Scale (Pelham et al., 1992). ARS = ADHD Rating Scale-5 (DuPaul et al., 2016). SSIS-RS = Social Skills Improvement System – Rating Scale (Gresham et al., 2011). RADS-2 = Reynolds Adolescents Depression Scale (Reynolds, 2002, 2008). SE = Self-Evaluation. SC = Somatic Complaints

Bivariate correlations reported using a Bonferroni adjusted alpha (α **=** 0.0004) are reported in Table 2. Both inattention and hyperactivity at T1 were significantly correlated with global social skills, cooperation, and responsibility at T2. Additionally, hyperactivity was significantly correlated with communication and self-control. Total social skills were significantly negatively correlated with depression at T3. Notably, ADHD symptom severity at T1 was not associated with depression scores at T3 (see supplementary information for the results of a baseline model of ADHD symptom severity at T1 predicting depression at T3 while controlling for covariates).

**Table 2:** Correlations between study variables

	1	2	3	4	5	6	7	8	9	10	11	12	13	14	15
1. Inattention (T1)															
2. Hyperactivity (T1)	0.48^***^														
3. Depression (T1)	0.01	− 0.04													
4. Depression (T3)	0.06	− 0.03	0.45^***^												
5. NSE (T3)	0.03	− 0.03	0.33^***^	0.89^***^											
6. Anhedonia	− 0.03	− 0.09	0.25^***^	0.57^***^	0.41^***^										
7. Dysphoric Mood	0.01	− 0.10	0.41^***^	0.89^***^	0.76^***^	0.33^***^									
8. SC	0.01	− 0.07	0.34^***^	0.84^***^	0.67^***^	0.23^**^	0.76^***^								
9. Social Skills (T2)	− 0.24^***^	− 0.23^***^	− 0.07	− 0.19^***^	− 0.22^*^	− 0.14^*^	− 0.11	−0.08							
10. Communication	− 0.13^*^	− 0.23^***^	− 0.02	− 0.13^*^	− 0.21^*^	− 0.13	− 0.10	−0.05	0.83^***^						
11. Cooperation	− 0.29^***^	− 0.30^***^	− 0.02	− 0.14^*^	− 0.18^*^	− 0.02	− 0.07	−0.07	0.82^***^	0.67^***^					
12. Assertion	− 0.11^*^	− 0.03	− 0.07	− 0.06	− 0.07	− 0.09	− 0.01	0.05	0.62^***^	0.50^***^	0.40^***^				
13. Responsibility	− 0.29^***^	− 0.30^***^	− 0.01	− 0.16^*^	− 0.18^*^	.−0.09	− 0.08	−0.06	0.82^***^	0.65^***^	0.79^***^	0.37^***^			
14. Empathy	− 0.19^**^	− 0.11^*^	− 0.02	− 0.17^*^	− 0.22^*^	− 0.06	− 0.11	− 0.09	0.82^***^	0.64^***^	0.66^***^	0.52^***^	0.68^***^		
15. Engagement	− 0.13^*^	− 0.06	− 0.12^*^	− 0.16^**^	− 0.17^*^	.−0.12	− 0.13	− 0.07	0.70^***^	0.61^***^	0.42^***^	0.51^***^	0.38^***^	0.52^***^	
16. Self-Control	− 0.14^*^	− 0.26^***^	− 0.03	− 0.10	− 0.16^*^	− 0.07	− 0.07	− 0.02	0.76^***^	0.59^***^	0.66^***^	0.36^***^	0.63^***^	0.56^***^	0.43^***^

ODD = Oppositional Defiant Disorder symptom severity. NSE = Negative Self-Evaluation. SC = Somatic Complaints. Social skills are total social skill scores. Higher scores on social skills and social skill factors indicate better social skills. Although we indicate significance value at *p* <.05, with the Bonferroni correction we adjusted thetype I error rate to 0.0004 as the threshold for statistical significance

*p <.05. **p <.001. ***p <.0004

**Fig. 1:**
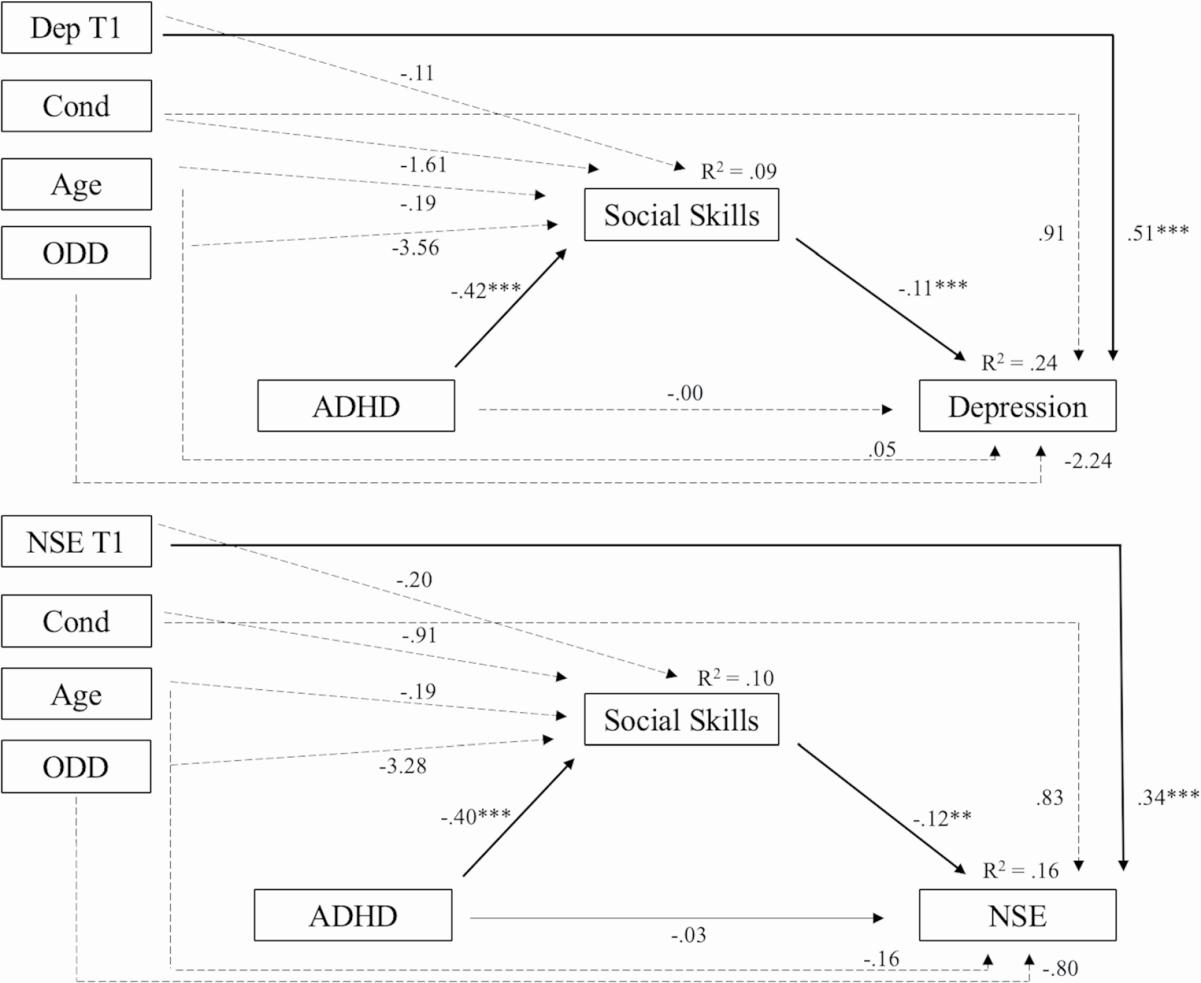
Mediation Models. *Note*. Cond = condition. Dep = Depression. NSE = Negative Self-Evaluation. ADHD symptom severity was measured at T1, social skills at T2 (one year after T1), and depression and NSE at T3 (1.5 years after baseline). Higher scores on social skills indicate better social skills. *p < .05. **p < .01. ***p < .001

Global social skills scores were entered as a mediating variable in the relation between ADHD symptom severity and depression, including the full sample of middle and high school students (see Fig. 1). The mediation analysis indicated that ADHD at T1 was a significant predictor of social skills at T2 (β = − 0.27, *b* **= −** 0.42, *SE* = 0.08, *t*(330) = −4.98, *p* <.001) and that social skills at T2 was a significant predictor of depression at T3 (β = − 0.17, *b* = − 0.11, *SE* = 0.03, *t*(329) = −3.38, *p* =.001). The direct effect of ADHD and depression was non-significant (*b* = − 0.00, *SE* = 0.05, t(329) = − 0.09, *p* =.93); however, the indirect effect was significant (*b* = 0.05, Boot *SE* = 0.02, Boot 95% CI [0.02, 0.08]; Completely Standardized: β = 0.05, Boot *SE* = 0.02, Boot 95% CI [0.02, 0.08]), suggesting that social skills mediated the relation between ADHD and depression.

Next, global social skills scores were entered as a mediating variable in the relation between ADHD symptom severity and each depression subscale (i.e., Negative Self-Evaluation, Anhedonia, Dysphoric Mood, and Somatic Complaints) in the middle school sample (*N* = 216). The mediation analyses indicated that, out of the four depression subscales, the indirect effect of social skills was only significant between ADHD and Negative Self-Evaluation (*b* = 0.05, Boot *SE* = 0.02, Boot 95% CI [0.01, 0.09]; Completely Standardized: β = 0.06, Boot *SE* = 0.02, Boot 95% CI [0.01, 0.11]). The direct effect of ADHD and Negative Self-Evaluation was non-significant (*b* = − 0.03, *SE* = 0.06, *t* (208) = −0.59, *p* =.56; see Table 3).

**Table 3:** Completely standardized indirect mediation effects of social skills between ADHD and depression subscales

RADS-2 Subscale	Effect	SE	LLCI	ULCI
NSE	0.06	0.02	0.0130	0.1089*
AN	0.04	0.02	− 0.0020	0.0781
DM	0.04	0.02	− 0.0019	0.0803
SC	0.03	0.02	− 0.0156	0.0706

RADS-2 = Reynolds Adolescent Depression Scale, Second Edition. NSE = Negative Self-Evaluation, AN = Anhedonia, DM = Dysphoric Mood. SC = Somatic Complaints

*Confidence interval does not include 0

Additionally, social skills subscale scores were entered as single mediators between ADHD symptom severity and depression. Among the seven subscale scores, Communication, Cooperation, Responsibility, and Empathy produced significant indirect effects (see Table 4).

**Table 4:** Completely standardized indirect mediation effects of social skills factors between ADHD and depression

Social Skill	Effect	SE	LLCI	ULCI
Communication	0.03	0.01	0.0033	0.0545*
Cooperation	0.05	0.02	0.0120	0.0889*
Assertion	0.00	0.01	− 0.0096	0.0127
Responsibility	0.05	0.02	0.0183	0.0959*
Empathy	0.03	0.01	0.0074	0.0533*
Engagement	0.01	0.01	− 0.0018	0.0303
Self-Control	0.02	0.01	− 0.0034	0.0470

*Confidence interval does not include 0

Finally, to consider the potential effect of age on our analyses we examined age as a moderator in our main model and found that it did not play a significant moderating role (age did not significantly interact with social skills functioning at T2 to predict depression at T3; *b* = 0.00, *SE* = 0.02, *p* =.813; index of moderated mediation: *b* = − 0.00, Boot *SE* = 0.01, 95% CI [−0.02, 0.01]).

## Discussion

We examined (1) social skills as a mediator of the relation between ADHD and depression, (2) social skills as a mediator of the relation between ADHD and depression facets (i.e., Negative Self-Evaluation, Anhedonia), and (3) types of social skills (i.e., Communication, Empathy) as mediators of the relation between ADHD and depression. Our results highlighted that social skills significantly contribute to the development of depression and, specifically, negative self-evaluation. This finding suggests that the impairment in social skills among adolescents with ADHD partially explains why they are at risk for developing depression. This study expanded on previous literature involving ADHD, social impairment, and depression in multiple ways. First, facets of depression, including negative self-evaluation, were examined as outcomes of ADHD, with social skills mediating the relation between ADHD and depression as well as between ADHD and negative self-evaluation. Self-evaluation is important to consider given that difficulties in sustaining attention are likely to impair adolescents’ self-worth (Mazzone et al., 2013) and that social impairment may explain this link. Second, to our knowledge, this study was the first to consider various social skill factors as mediators between ADHD and depression. This investigation allowed for a deeper understanding of what areas of social functioning can most explain the relation between ADHD and depression. It also provided insight into which social skills interventions may want to target to prevent depression. Lastly, we evaluated the mediation models across a large age range of 11- to 17-year-old, enhancing the generalizability of our results.

### Facets of Depression

Considering what areas of depression are most predicted by ADHD may inform prevention efforts targeting the onset of depression. This study found that, out of the four facets that contribute to adolescent depression (anhedonia, negative self-evaluation, dysphoric mood, and somatic complaints), social skills only significantly mediated the relation between ADHD and negative self-evaluation. The sample for this analysis consisted of middle school students, so this finding aligns with previous research suggesting that low self-worth often occurs before the onset of depression and may lead to the development of depression in later adolescence (Lewinsohn et al., 1994; Orth et al., 2008). Further, previous studies found that adolescents with ADHD are at risk for developing low self-worth (Mazzone et al., 2013) and that social impairment is associated with one’s beliefs about oneself (Becker et al., 2017; Scharf & Mayseless, 2009). Therefore, this finding may inform prevention efforts by highlighting the importance of targeting the development of negative self-evaluation in young adolescents with ADHD, as negative self-evaluation may be the area of depression most impacted by social skills impairment.

### Social Skill Factors

Additionally, this study was the first to examine various specific social skill factors (e.g., responsibility, cooperation, communication) as mediators between ADHD and depression. By evaluating specific types of social skills, we were able to understand further what areas of social impairment can most explain the development of depression in adolescents with ADHD and, thus, could be beneficial areas to target in interventions to prevent depression. Previous research has supported the strong mediating role of peer relationships between ADHD and depression (Humphreys et al., 2013; Powell et al., 2020; Roy et al., 2015); therefore, we hypothesized that the social skills related to friendships (i.e., cooperation, empathy, engagement, and communication) would mediate the relation between ADHD and depression, while the other social skills (i.e., assertion, self-control, responsibility) would not.

Our hypotheses were partially supported, as we found that communication, cooperation, empathy, and responsibility were significant mediators. This finding aligns with theoretical and empirical support that communication, cooperation, and empathy are negatively linked with depression, which is likely explained by these social skills’ impact on friendships. However, it remains unclear why responsibility may have a prominent mediating role between ADHD and depression, although some research suggests that personal responsibility is related to self-worth in adolescence and, therefore, may be particularly related to depression (Mergler et al., 2007).

### ADHD and Depression

An unexpected result was found in the bivariate correlations indicating that ADHD symptom severity at T1 was not associated with depression at T1 or T3. This result was surprising since inattention is a symptom of major depressive disorder (American Psychiatric Association, 2022), and numerous studies have found an association between ADHD and depression (Daviss, 2008; Meinzer et al., 2013, 2016). It is possible that the lack of association between ADHD and depression could be a result of range restriction, given that our sample exhibited mostly high ADHD symptom severity (all participants were diagnosed with ADHD) and relatively low depression. Another explanation of the significant indirect mediation effect with a non-significant direct effect is that there may be some outcomes of ADHD, such as impaired social skills, that act as a risk factor for depression, with others (e.g., social acceptance) acting as protective factors against the development of depression for adolescents with ADHD (Dvorsky & Langberg, 2016). Although social skills impairment is a common aspect of ADHD, research suggests that not all adolescents with ADHD will experience it (Lee et al., 2008; Modesto-Lowe et al., 2011; Ray et al., 2017).

It is possible that the protective factors that some adolescents with ADHD develop, such as social acceptance, may counter the risk of developing depression and, thus, explain why a direct effect of ADHD on depression was not observed in our study. Future research is needed to investigate this hypothesis.

Given the lack of correlation between these variables, one might express concern over conducting mediation analyses with them. Although there have been recommendations to test the relation between the X and Y variables of a mediation model as a requirement for mediation analysis, guidelines suggest that this test may not be necessary when there is theoretical support for the relation between X and Y and a small effect size is expected (Shrout & Bolger, 2002). Since these conditions are met with ADHD and depression, it remains useful and informative to test variables that may explain their relation, such as social impairment.

### Limitations and Future Directions

Despite the numerous strengths of the present study, including a sample with a broad age range that consisted of participants who were diagnosed with ADHD and in which the mediation constructs were examined longitudinally over 1.5 years, there are also notable limitations. First, our sample lacked representation from Asian and Native American/Alaska Native groups compared to the U.S. population (U.S. Census Bureau, 2023). Given this limitation, it is possible that our results will not apply to all races, and it remains unclear how race could impact our results. Because this study did not test how race may impact the mediation models, future research may be needed to examine the role of race in the models we tested.

Second, assertion had low reliability in our sample, with a Cronbach’s alpha of 0.68 in the middle school students and 0.57 in the high school students. Although assertion has been found to have the lowest reliability out of the other SSIS factors (α = 0.77; Gresham et al., 2011), data from our sample had a particularly low internal consistency. Therefore, our finding that assertion did not significantly mediate the relationships between ADHD and depression should be interpreted with caution.

Third, because the high school sample completed the short form of the RADS-2, we could not examine the facets of depression (e.g., negative self-evaluation, anhedonia) in this age group. Future research should examine whether the prominent outcome of negative self-evaluation is upheld with older adolescents. Another limitation of the present study is that the RADS-2 scores were in the non-clinical range (T-scores were less than 60). This finding was surprising given the extant literature suggesting that adolescents with ADHD are at risk for developing depression (Daviss, 2008; Meinzer et al., 2013). This finding potentially limited our ability to evaluate depression symptom severity as an outcome, as there was a limited range of RADS-2 scores.

Fourth, our sample included a broad age range, so it is possible that our results may differ across ages. Future research should examine the extent to which social skills functioning plays a more prominent role in the development of depression in older adolescents than children with ADHD. However, to preliminarily address this question, we examined age as a moderator in our main model and found that it did not play a significant moderating role. Thus, while our preliminary analysis suggests that age does not significantly impact the extent to which social skills mediate ADHD symptom severity and the development of depression, future research should formally investigate this hypothesis with a more well-balanced sample across grade levels.

Another limitation of the present study was sole reliance of parent report of social skills. There are various other aspects and considerations of social functioning, including peer conflict, peer rejection, and teacher-reported social skills. Prior studies found peer conflict and peer rejection to also be mediators of ADHD and depression (Humphreys et al., 2013; Powell et al., 2020; Roy et al., 2015). These aspects of social functioning are important to consider, and future research may consider whether there are differences in how these aspects of social functioning play a role in the development of depression.

In our sample, all adolescents were diagnosed with ADHD, so we could not compare the mediation models between those with and without ADHD. Therefore, one area of future research could be to investigate which social skills predict depression in youth without ADHD and how negative self-evaluation differs between those with ADHD and those without. Particularly, it may be of interest to explore which social skills factors are significant predictors between ADHD and non-ADHD groups. This topic could be of importance in establishing whether the ability of social skills to contribute to the development of depression and negative self-evaluation is specific to those with ADHD.

Lastly, although we used Bonferroni corrections when interpreting the correlation table, we did not use it when investigating the four facets of depression as outcomes and seven social skill factors as mediators as this approach was too conservative for our second research question intended to generate hypotheses regarding types of depression and social skills that are primary to the relationships being studied. Therefore, these results need to be interpreted with caution, as we consider them to be exploratory findings. However, our findings raise hypotheses about (1) the development of negative self-evaluation in adolescents with ADHD and (2) which social skills matter most in the development of depression. Future research could replicate and expand on the roles of communication, cooperation, empathy, and responsibility.

### Implications

In summary, our results suggest that social skills needed for the development and continuity of friendships, including communication, empathy, and cooperation, may be the most important in the development of depression in adolescents with ADHD. Additionally, our results suggest that, in younger adolescents (grades 6–8), negative self-evaluation is likely the most prominent outcome of ADHD, with social skills mediating their relation. These results highlight that early, targeted social skills intervention, particularly those that promote peer connectedness and, in turn, boost positive self-concept, may be beneficial for adolescents with ADHD. Importantly, these results offer a potential avenue for reducing depression risk among adolescents with ADHD, especially when implemented during early adolescence, as it has appeared to be a critical developmental window. Although numerous social skills training programs have been developed for youth with ADHD, research suggest that the effects of these interventions are often modest and improvements do not always generalize beyond structured settings (Evans et al., 2018). However, recent interventions utilize peer involvement, parent engagement, and training strategies (Capps et al., 2024; Evans et al., 2024; Pfiffner et al., 2018), which may be adapted to help with promoting change in social functioning domains (i.e., social skills). Our findings, which highlight specific social skills as central to the relation between ADHD and depression, may help refine these interventions by identifying the specific social skills most relevant to reducing depression risk.

## Supplementary Information

Below is the link to the electronic supplementary material.

Supplementary Material 1

## Data Availability

The data generated during and/or analyzed during the current study are available on reasonable request.

## References

[CR1] Ágrez, K., Visky, Z., Hámori, G., Takács, M., Pulay, A. J., Réthelyi, J. M., & Bunford, N. (2025). Not just old wine in new bottles: Polygenic liability for ADHD is associated with electrophysiological affective-motivational processing beyond anxiety, depression, and ODD. *Translational Psychiatry,**15*(1), Article 213. 40555725 10.1038/s41398-025-03434-z PMC12187935

[CR2] American Psychiatric Association (2022). *Diagnostic and statistical manual of mental disorders* (5th ed., text rev.).

[CR3] Arnold, L. E., Hodgkins, P., Kahle, J., Madhoo, M., & Kewley, G. (2020). Long-term outcomes of ADHD: Academic achievement and performance. *Journal of Attention Disorders,**24*(1), 73–85. 25583985 10.1177/1087054714566076

[CR4] Bagwell, C. L., Molina, B. S., Pelham, W. E., & Hoza, B. (2001). Attention-deficit hyperactivity disorder and problems in peer relations: Predictions from childhood to adolescence. *Journal of the American Academy of Child and Adolescent Psychiatry,**40*(11), 1285–1292. 11699802 10.1097/00004583-200111000-00008

[CR5] Becker, S. P., Langberg, J. M., Evans, S. W., Girio-Herrera, E., & Vaughn, A. J. (2015). Differentiating anxiety and depression in relation to the social functioning of young adolescents with ADHD. *Journal of Clinical Child & Adolescent Psychology,**44*(6), 1015–1029. 25010226 10.1080/15374416.2014.930689 PMC4289476

[CR6] Becker, S. P., Mehari, K. R., Langberg, J. M., & Evans, S. W. (2017). Rates of peer victimization in young adolescents with ADHD and associations with internalizing symptoms and self-esteem. *European Child & Adolescent Psychiatry,**26*, 201–214. 27315106 10.1007/s00787-016-0881-y PMC6048591

[CR7] Biederman, J., Ball, S. W., Monuteaux, M. C., Mick, E., Spencer, T. J., McCreary, M., Cote, M., & Faraone, S. V. (2008). New insights into the comorbidity between ADHD and major depression in adolescent and young adult females. *Journal of the American Academy of Child & Adolescent Psychiatry,**47*(4), 426–434. 18388760 10.1097/CHI.0b013e31816429d3

[CR8] Blackman, G. L., Ostrander, R., & Herman, K. C. (2005). Children with ADHD and depression: A multisource, multimethod assessment of clinical, social, and academic functioning. *Journal of Attention Disorders,**8*(4), 195–207. 16110050 10.1177/1087054705278777

[CR9] Brown, J. D., Dutton, K. A., & Cook, K. E. (2001). From the top down: Self-esteem and self-evaluation. *Cognition and Emotion*, *15*(5), 615–631.

[CR10] Buhrmester, D. (1990). Intimacy of friendship, interpersonal competence, and adjustment during preadolescence and adolescence. *Child Development*, *61*(4), 1101–1111. 2209180

[CR11] Capaldi, D. M. (1992). Co-occurrence of conduct problems and depressive symptoms in early adolescent boys: II. A 2-year follow-up at Grade 8. *Development and Psychopathology,**4*(1), 125–144. 10.1017/s0954579499001959 10208356

[CR12] Capps, R. E., Evans, S. W., Owens, J. S., & Allan, D. M. (2024). A peer-supported school engagement intervention for youth with attention problems: Development and implementation. *School Mental Health,**16*(3), 649–666.

[CR14] Daviss, W. B. (2008). A review of co-morbid depression in pediatric ADHD: Etiologies, phenomenology, and treatment. *Journal of Child and Adolescent Psychopharmacology,**18*, 565–571. 19108661 10.1089/cap.2008.032 PMC2699665

[CR15] DuPaul, G. J., Reid, R., Anastopoulos, A. D., Lambert, M. C., Watkins, M. W., & Power, T. J. (2016). Parent and teacher ratings of attention-deficit/hyperactivity disorder symptoms: Factor structure and normative data. *Psychological Assessment,**28*(2), 214–225. 26011476 10.1037/pas0000166

[CR16] Dvorsky, M. R., & Langberg, J. M. (2016). A review of factors that promote resilience in youth with ADHD and ADHD symptoms. *Clinical Child and Family Psychology Review,**19*(4), 368–391. 27747466 10.1007/s10567-016-0216-z

[CR17] Dvorsky, M. R., Langberg, J. M., Becker, S. P., & Evans, S. W. (2019). Trajectories of global self-worth in adolescents with ADHD: Associations with academic, emotional, and social outcomes. *Journal of Clinical Child & Adolescent Psychology,**48*(5), 765–780. 29714502 10.1080/15374416.2018.1443460 PMC6287970

[CR18] Eadeh, M., Bourchtein, E., Langberg, J. M., Eddy, L. D., Oddo, L., Molitor, S. J., & Evans, S. W. (2017). Longitudinal evaluation of the role of academic and social impairment and parent-adolescent conflict in the development of depression in adolescents with ADHD. *Journal of Child and Family Studies,**26*(9), 2374–2385. 29713135 10.1007/s10826-017-0768-7 PMC5916842

[CR19] Eberhart, N. K., & Hammen, C. L. (2006). Interpersonal predictors of onset of depression during the transition to adulthood. *Personal Relationships,**13*(2), 195–206.

[CR20] Evans, S. W., Allen, J., Moore, S., & Strauss, V. (2005). Measuring symptoms and functioning of youth with ADHD in middle schools. *Journal of Abnormal Child Psychology,**33*(6), 695–706. 16328745 10.1007/s10802-005-7648-0

[CR23] Evans, S. W., DuPaul, G. J., Benson, K., Owens, J. S., Fu, Q., Cleminshaw, C., Kipperman, K., & Margherio, S. (2024). Social functioning outcomes of a high school-based treatment program for adolescents with ADHD. *Journal of Clinical Child & Adolescent Psychology,**53*(3), 413–428. 37494306 10.1080/15374416.2023.2235693

[CR21] Evans, S. W., Langberg, J. M., Schultz, B. K., Vaughn, A., Altaye, M., Marshall, S. A., & Zoromski, A. K. (2016). Evaluation of a school-based treatment program for young adolescents with ADHD. *Journal of Consulting and Clinical Psychology,**84*(1), 15–30. 26501496 10.1037/ccp0000057

[CR22] Evans, S. W., Owens, J. S., Wymbs, B. T., & Ray, A. R. (2018). Evidence-based psychosocial treatments for children and adolescents with attention deficit/hyperactivity disorder. *Journal of Clinical Child & Adolescent Psychology,**47*(2), 157–198. 29257898 10.1080/15374416.2017.1390757

[CR24] Gardner, D. M., & Gerdes, A. C. (2015). A review of peer relationships and friendships in youth with ADHD. *Journal of Attention Disorders,**19*(10), 844–855. 24062277 10.1177/1087054713501552

[CR25] Gresham, F. M., & Elliott, S. N. (1990). *The Social Skills Rating System*. American Guidance Service.

[CR26] Gresham, F. M., & Elliott, S. N. (2008). *Social Skills Improvement System: Rating Scales*. Pearson Assessments.

[CR27] Gresham, F., Elliott, S., Vance, M., & Cook, C. (2011). Comparability of the social skills rating system to the social skills improvement system: Content and psychometric comparisons across elementary and secondary age levels. *School Psychology Quarterly*, *26*(1), 27–44.

[CR28] Harter, S. (2012). Self-perception profile for adolescents: Manual and questionnaires. *Denver CO: Univeristy of Denver Department of Psychology,* 31–45.

[CR29] Hayes, A. F. (2022). *Introduction to mediation, moderation, and conditional process analysis: A regression-based approach* (3rd edition). The Guilford Press.

[CR30] Herman, K. C., Lambert, S. F., Ialongo, N. S., & Ostrander, R. (2007). Academic pathways between attention problems and depressive symptoms among urban African American children. *Journal of Abnormal Child Psychology,**35*(2), 265–274. 17211727 10.1007/s10802-006-9083-2 PMC3674873

[CR31] Holsen, I., Kraft, P., & Vittersø, J. (2000). Stability in depressed mood in adolescence: Results from a 6-year longitudinal panel study. *Journal of Youth and Adolescence*, *29*(1), 61–78.

[CR32] Humphreys, K. L., Katz, S. J., Lee, S. S., Hammen, C., Brennan, P. A., & Najman, J. (2013). The association of ADHD and depression: Mediation by peer problems and parent–child difficulties in two complementary samples. *Journal of Abnormal Psychology,**122*, 854–867. 24016021 10.1037/a0033895 PMC3806877

[CR33] King, C. A., Naylor, M. W., Segal, H. G., Evans, T., & Shain, B. N. (1993). Global self-worth, specific self-perceptions of competence, and depression in adolescents. *Journal of the American Academy of Child and Adolescent Psychiatry,**32*(4), 745–752. 8340294 10.1097/00004583-199307000-00007

[CR34] Klein, D. N., Kotov, R., & Bufferd, S. J. (2011). Personality and depression: Explanatory models and review of the evidence. *Annual Review of Clinical Psychology,**7*, 269–295. 21166535 10.1146/annurev-clinpsy-032210-104540 PMC3518491

[CR35] Knouse, L. E., Ziegler, M., Lavine, I., Zhang, J., Cheng, Y., & Ul Ain, H. (2023). Avoidant automatic thoughts are associated with task avoidance and inattention in the moment. *Cognitive Therapy and Research,**48*, 866–879.

[CR36] Lee, S. S., Lahey, B. B., Owens, E. B., & Hinshaw, S. P. (2008). Few preschool boys and girls with ADHD are well-adjusted during adolescence. *Journal of Abnormal Child Psychology,**36*(3), 373–383. 17914666 10.1007/s10802-007-9184-6

[CR37] Lewinsohn, P. M., Clarke, G. N., Seeley, J. R., & Rohde, P. (1994). Major depression in community adolescents: Age at onset, episode duration, and time to recurrence. *Journal of the American Academy of Child and Adolescent Psychiatry,**33*(6), 809–818. 7598758 10.1097/00004583-199407000-00006

[CR38] Mannarino, A. P. (1976). Friendship patterns and altruistic behavior in preadolescent males. *Developmental Psychology,**12*(6), 555–556.

[CR39] Marshall, S. L., Ciarrochi, J., Parker, P. D., & Sahdra, B. K. (2019). Is self-compassion selfish? The development of self-compassion, empathy, and prosocial behavior in adolescence. *Journal of Research on Adolescence,**30*, 472–484. 30884003 10.1111/jora.12492

[CR40] Mazzone, L., Postorino, V., Reale, L., Guarnera, M., Mannino, V., Armando, M., Fatta, L., De Peppo, L., & Vicari, S. (2013). Self-esteem evaluation in children and adolescents suffering from ADHD. *Clinical Practice and Epidemiology in Mental Health,**9*, 96–102. 23878614 10.2174/1745017901309010096 PMC3715757

[CR41] McQuade, J. D., Hoza, B., Murray-Close, D., Waschbusch, D. A., & Owens, J. S. (2011). Changes in self-perceptions in children with ADHD: A longitudinal study of depressive symptoms and attributional style. *Behavior Therapy*, *42*(2), 170–182. 21496504 10.1016/j.beth.2010.05.003 PMC3990436

[CR42] Meinzer, M. C., Lewinsohn, P., Pettit, J., Seeley, J., Gau, J., Chronis-Tuscano, A., & Waxmonsky, J. (2013). Attention-deficit/hyperactivity disorder in adolescence predicts onset of major depressive disorder through early adulthood. *Depression and Anxiety,**30*(6), 546–553. 23424020 10.1002/da.22082 PMC3788356

[CR43] Meinzer, M., Pettit, J., Waxmonsky, J., Gnagy, E., Molina, B., & Pelham, W. (2016). Does childhood attention-deficit/hyperactivity disorder predict levels of depressive symptoms during emerging adulthood? *Journal of Abnormal Child Psychology*, *44*, 787–797. 26272531 10.1007/s10802-015-0065-0 PMC4754165

[CR44] Mergler, A., Spencer, F. H., & Patton, W. (2007). Relationships between personal responsibility, emotional intelligence, and self-esteem in adolescents and young adults. *The Australian Educational and Developmental Psychologist*, *24*(1), 5–18.

[CR45] Milfont, T. L., Merry, S., Robinson, E., Denny, S., Crengle, S., & Ameratunga, S. (2008). Evaluating the short form of the Reynolds Adolescent Depression Scale in New Zealand adolescents. *Australian and New Zealand Journal of Psychiatry,**42*(11), 950–954. 18941959 10.1080/00048670802415343

[CR46] Miller, G. A., & Lazowski, L. E. (2001). *The adolescent SASSI-A2 manual: Identifying substance use disorders.* SASSI Institute.

[CR47] Modesto-Lowe, V., Yelunina, L., & Hanjan, K. (2011). Attention-deficit/hyperactivity disorder: A shift toward resilience? *Clinical Pediatrics*, *50*(6), 518–524. 21262756 10.1177/0009922810394836

[CR48] Molina, B. S. G., Pelham, W. E., Blumenthal, J., & Galiszewski, E. (1998). Agreement among teachers’ behavior ratings of adolescents with a childhood history of attention deficit hyperactivity disorder. *Journal of Clinical Child Psychology,**27*(3), 330–339. 9789192 10.1207/s15374424jccp2703_9 PMC4871601

[CR49] Morgan, J. E., Dvorsky, M. R., Meza, J. I., Schumacher, L. T., & Pfiffner, L. J. (2022). Co-occurring psychopathology moderates social skills improvement in a randomized controlled trial of a collaborative school-home intervention for children with ADHD. *Journal of Clinical Child & Adolescent Psychology,**51*(4), 543–555. 32930610 10.1080/15374416.2020.1815206 PMC7956906

[CR50] Mrug, S., Molina, B. S. G., Hoza, B., Gerdes, A. C., Hinshaw, S. P., Hechtman, L., & Arnold, L. E. (2012). Peer rejection and friendships in children with attention-deficit/hyperactivity disorder: Contributions to long-term outcomes. *Journal of Abnormal Child Psychology,**40*(6), 1013–1026. 22331455 10.1007/s10802-012-9610-2 PMC3384771

[CR51] Orth, U., Robins, R. W., & Roberts, B. W. (2008). Low self-esteem prospectively predicts depression in adolescence and young adulthood. *Journal of Personality and Social Psychology,**95*(3), 695–708. 18729703 10.1037/0022-3514.95.3.695

[CR52] Ortuño-Sierra, J., Aritio-Solana, R., Inchausti, F., Luis, ECde, Molina, B. L., Albéniz, APde, & Fonseca-Pedrero, E. (2017). Screening for depressive symptoms in adolescents at school: New validity evidences on the short form of the Reynolds Depression Scale. *PLoS ONE,**12*(2), Article e0170950. 28222193 10.1371/journal.pone.0170950 PMC5319653

[CR53] Pelham, W. E., Evans, S. W., Gnagy, E., & Greenslade, K. (1992). Teacher ratings of *DSM-III-R* symptoms for disruptive behavior disorders: Prevalence, factor analyses, and conditional probabilities in a special education sample. *School Psychology Review,**21*, 285–299.

[CR54] Pfiffner, L. J., Rooney, M. E., Jiang, Y., Haack, L. M., Beaulieu, A., & McBurnett, K. (2018). Sustained effects of collaborative school-home intervention for attention-deficit/hyperactivity disorder symptoms and impairment. *Journal of the American Academy of Child & Adolescent Psychiatry,**57*(4), 245–251. 29588050 10.1016/j.jaac.2018.01.016

[CR55] Platt, B., Cohen Kadosh, K., & Lau, J. Y. (2013). The role of peer rejection in adolescent depression. *Depression and Anxiety,**30*(9), 809–821. 23596129 10.1002/da.22120

[CR56] Powell, V., Riglin, L., Hammerton, G., Eyre, O., Martin, J., Anney, R., Thapar, A., & Rice, F. (2020). What explains the link between childhood ADHD and adolescent depression? Investigating the role of peer relationships and academic attainment. *European Child & Adolescent Psychiatry*, *29*(11), 1581–1591. 31932968 10.1007/s00787-019-01463-w PMC7595988

[CR57] Ray, A. R., Evans, S. W., & Langberg, J. M. (2017). Factors associated with healthy and impaired social functioning in young adolescents with ADHD. *Journal of Abnormal Child Psychology*, *45*, 883–897. 27796691 10.1007/s10802-016-0217-x PMC5409909

[CR58] Reynolds, W. M. (2002). *Reynolds Adolescent Depression Scale* (2nd ed.). Wiley.

[CR59] Reynolds, W. M. (2008). *Reynolds Adolescent Depression Scale* (2nd ed., Short Form [RADS-2: SF]). Psychological Assessment Resources.

[CR60] Riglin, L., Leppert, B., Dardani, C., Thapar, A. K., Rice, F., O’Donovan, M. C., Davey Smith, G., Stergiakouli, E., Tilling, K., & Thapar, A. (2021). ADHD and depression: Investigating a causal explanation. *Psychological Medicine,**51*(11), 1890–1897. 32249726 10.1017/S0033291720000665 PMC8381237

[CR61] Roy, A., Hartman, C. A., Veenstra, R., & Oldehinkel, A. J. (2015). Peer dislike and victimisation in pathways from ADHD symptoms to depression. *European Child & Adolescent Psychiatry*, *24*(8), 887–895. 25348085 10.1007/s00787-014-0633-9

[CR62] Scharf, M., & Mayseless, O. (2009). Socioemotional characteristics of elementary school children identified as exhibiting social leadership qualities. *The Journal of Genetic Psychology*, *170*(1), 73–96. 19230521 10.3200/GNTP.170.1.73-96

[CR63] Schoeler, T., Duncan, L., Cecil, C. M., Ploubidis, G. B., & Pingault, J. B. (2018). Quasi-experimental evidence on short- and long-term consequences of bullying victimization: A meta-analysis. *Psychological Bulletin,**144*(12), 1229–1246. 30475016 10.1037/bul0000171

[CR64] Shorey, S., Ng, E. D., & J. Wong, C. H. (2022). Global prevalence of depression and elevated depressive symptoms among adolescents: A systematic review and meta-analysis. *British Journal of Clinical Psychology,**61*(2), 287–305. 34569066 10.1111/bjc.12333

[CR65] Shrout, P. E., & Bolger, N. (2002). Mediation in experimental and nonexperimental studies: New procedures and recommendations. *Psychological Methods,**7*(4), 422–445. 12530702

[CR66] Sibley, M. H., Molina, B. S., Gnagy, E. M., Waschbusch, D. A., Garefino, A. C., Kuriyan, A. B., Babinski, D. E., & Karch, K. M. (2012). Diagnosing ADHD in adolescence. *Journal of Consulting and Clinical Psychology,**80*(1), 139–150. 22148878 10.1037/a0026577 PMC4085687

[CR67] Slomkowski, C., Klein, R. G., & Mannuzza, S. (1995). Is self-esteem an important outcome in hyperactive children? *Journal of Abnormal Child Psychology*, *23*(3), 303–315. 7642839 10.1007/BF01447559

[CR68] Treuting, J. J., & Hinshaw, S. P. (2001). Depression and self-esteem in boys with attention-deficit/hyperactivity disorder: Associations with comorbid aggression and explanatory attributional mechanisms. *Journal of Abnormal Child Psychology,**29*(1), 23–39. 11316333 10.1023/a:1005247412221

[CR69] Turgay, A., Ansari, R., Schwartz, M., et al. (2005). *Comorbidity differences in ADHD throughout the life cycle [Paper presentation]. Scientific and clinical report session*. American Psychiatric Association Annual Scientific Meeting.

[CR13] U.S. Census Bureau, Population Division. (2023). *Projected Population Distribution by Race and Hispanic Origin for the United States, Main Series: 2022–2060*. Retrieved February 7, 2025. https://www.census.gov/data/tables/2023/demo/popproj/2023-summary-tables.html

[CR70] Wechsler, D. (2003). *Wechsler Intelligence Scale for Children, Fourth Edition (WISC-IV)*. The Psychological Cooperation.

[CR71] Wechsler, D. (2011). *Wechsler Abbreviated Scale of Intelligence-2nd Edition* (NCS Pearson).

[CR72] Weller, E. B., Weller, R. A., Fristad, M. A., Rooney, M. T., & Schecter, J. (2000). Children’s interview for psychiatric syndromes (ChIPS). *Journal of the American Academy of Child & Adolescent Psychiatry,**39*(1), 76–84. 10638070 10.1097/00004583-200001000-00019

[CR73] Willcutt, E. G., Nigg, J. T., Pennington, B. F., Solanto, M. V., Rohde, L. A., Tannock, R., Loo, S. K., Carlson, C. L., McBurnett, K., & Lahey, B. B. (2012). Validity of DSM-IV attention deficit/hyperactivity disorder symptom dimensions and subtypes. *Journal of Abnormal Psychology,**121*(4), 991–1010. 22612200 10.1037/a0027347 PMC3622557

[CR74] Wright, K. D., Waschbusch, D. A., & Frankland, B. W. (2007). Combining data from parent ratings and parent interview when assessing ADHD. *Journal of Psychopathology and Behavioral Assessment,**29*, 141–148.

[CR75] Zoromski, A., Owens, J. S., Evans, S. W., & Brady, C. (2015). Identifying ADHD symptoms most associated with impairment in early childhood, middle childhood, and adolescence using teacher report. *Journal of Abnormal Child Psychology,**43*, 1243–1255. 25899878 10.1007/s10802-015-0017-8

