# Supplementary material for: Linking ADHD to Depression in Adolescents: the Mediating Role of Social Skills
Source: Res Child Adolesc Psychopathol. 2026 Mar 2;54(2):40. doi: 10.1007/s10802-026-01430-5 (PMC12953414; doi:10.1007/s10802-026-01430-5)
Supplement: Supplementary file 1 — Supplementary Material 1 [file 10802_2026_1430_MOESM1_ESM.docx]

**Table S1**

*Regression Analysis Predicting Depression at T3*

|  | *B* | *SE B* | *β* | *t* | *p* |
| --- | --- | --- | --- | --- | --- |
| Intercept | 17.97 | 5.54 |  | 3.24 | .001 |
| ADHD T1 | 0.04 | 0.05 | .04 | 0.83 | .410 |
| Depression T1 | 0.52 | 0.06 | .46 | 9.29 | <.001 |
| Treatment Condition | 1.09 | 0.71 | .08 | 1.53 | .128 |
| Age | 0.07 | 0.34 | .01 | 0.21 | .831 |
| ODD Diagnosis | -1.84 | 1.35 | -.07 | -1.37 | .173 |
